# ErgoReport: A Holistic Posture Assessment Framework Based on Inertial Data and Deep Learning

**DOI:** 10.3390/s25072282

**Published:** 2025-04-03

**Authors:** Diogo R. Martins, Sara M. Cerqueira, Ana Pombeiro, Alexandre Ferreira da Silva, Ana Maria A. C. Rocha, Cristina P. Santos

**Affiliations:** 1 Center for MicroElectroMechanical Systems (CMEMS), University of Minho, 4800-058 Guimarães, Portugal; id9484@alunos.uminho.pt (S.M.C.); asilva@dei.uminho.pt (A.F.d.S.); 2 Robert Bosch GmbH, 70469 Stuttgart, Germany; ana.pombeiro@pt.bosch.com; 3 LABBELS–Associate Laboratory, University of Minho, 4800-058 Guimarães, Portugal; 4 ALGORITMI Research Centre, Universidade do Minho, 4710-057 Braga, Portugal; arocha@dps.uminho.pt

**Keywords:** deep learning, ergonomic risk assessment, inertial-based posture recognition, posture monitoring, work-related musculoskeletal disorders

## Abstract

Awkward postures are a significant contributor to work-related musculoskeletal disorders (WRMSDs), which represent great social and economic burdens. Various posture assessment tools assess WRMSD risk but fall short in providing an elucidating risk breakdown to expedite the typical time-consuming ergonomic assessments. Quantifying, automating, but also complementing posture risk assessment become crucial. Thus, we developed a framework for a holistic posture assessment, able to, through inertial data, quantify the ergonomic risk and also qualitatively identify the posture leading to it, using Deep Learning. This innovatively enabled the generation of a report in a graphical user interface (GUI), where the ergonomic score is intuitively associated with the postures adopted, empowering workers to learn which are the riskiest postures, and helping ergonomists and managers to redesign critical work tasks. The continuous posture assessment also considered the previous postures’ impact on joint stress through a kinematic wear model. As use case, thirteen subjects replicated harvesting and bricklaying, work tasks of the two activity sectors most affected by WRMSDs, agriculture and construction, and a posture assessment was conducted. Three ergonomists evaluated this report, considering it very useful in improving ergonomic assessments’ effectiveness, expeditiousness, and ease of use, with the information easily understandable and reachable.

## 1. Introduction

Musculoskeletal disorders (MSDs) represent 60% of the work-related health problems in the European Union [1]. The sixth wave of the European Working Conditions Survey revealed that, in 2015, 54% of the Portuguese working population suffered from one or more MSDs in the previous 12 months [1]. Despite the economy’s advances towards automation, many jobs still consist of risky and physically demanding tasks [2]. Working in regular or sustained awkward postures constitutes a risk factor for developing work-related musculoskeletal disorder (WRMSDs) [3]. Their consequences are associated with work-limiting pain that decreases psychological health, job satisfaction, and productivity, and it may lead to worker absenteeism or even early retirement [1,3]. MSDs were responsible for 60% of permanent work incapacity [4] and, in 2015, 53% of the workers with WRMSDs reported work absences [1]. The total costs of WRMSDs are estimated at up to 2% of the gross domestic product of the European Union (EUR 240 billion) [4].

Agriculture and construction are the leading activity sectors in WRMSDs’ reports, with prevalences up to, respectively, 60% (for backache) and 54% (for upper limbs) [1]. Regarding the agriculture sector, harvesting workers reported a WRMSDs’ prevalence of 86% during one year [5]. In 2015, among the construction sector’s workers, excessive effort from lifting and lowering caused 30% of the WRMSDs, while pushing, pulling, holding, carrying, and catching were responsible for 37% [6]. Particularly, bricklayers spend 93% of their working time bending, twisting, and performing repetitive motions [6].

Taking proper preventive measures can lower the risk of WRMSDs. Indeed, MSDs have proven to be less frequent when an ergonomic risk analysis that leads to the adoption of interventions is conducted [7].

There are a variety of tools available for WRMSDs’ risk assessment. The most used ones are based on observation, conducted by an expert. However, most ergonomic tools are based on sampled snapshot assessments of the postures sustained by the worker throughout their work shift. These methods are very time-consuming, depend on the ergonomist’s experience, and disregard the impact of previous postures on the actual risk [8]. Furthermore, since typical ergonomic assessment tools are based on the ergonomists’ observations, they were designed to be practical and easy to apply, resulting in simple methods that, by themselves, do not convey an explanatory risk analysis. For an objective and reliable ergonomic assessment, sensor-based measurements are required [3]. Nonetheless, these direct methods can acquire a substantial amount of data, and providing too much information can make the analysis cumbersome. Intuitive and expeditious manners are required to display sensor-based information.

Every job consists of a series of tasks with a certain goal [9], whose accomplishment requires a series of postures. By decomposing the work tasks into postures, a more detailed risk assessment is possible. For example, one can determine the critical points that need to be modified in the task [9] and can assess each posture class separately, providing greater comprehensibility and allowing the improvement in ergonomics [9], while considering productivity and the task goal. Withal, determining those postures’ occurrences is challenging since a manual recording, by the worker or the ergonomist, of the sequence of postures is neither practical nor feasible. Lately, automated posture recognition has been gaining importance in context-aware systems in several domains, such as health and ambient assisted living [10]. These methods facilitate continuous monitoring and can be a solution. Artificial Intelligence (AI) solutions are more appropriate than other automated approaches (e.g., finite-state machines) for activity or posture recognition, due to the considerable complexity associated with the number of body segments involved and intra- and inter-subject variability. AI models encompass traditional Machine Learning (ML) algorithms, which primarily depend on domain knowledge to wisely design features [11,12]. Deep Learning (DL) is a type of ML, in which features are automatically extracted in multiple layers of the learning models, deep neural networks, fed with raw input data [13]. Particularly, DL approaches have become more used, and they have outperformed ML in many applications, overcoming the ML’s limitation of handcrafted feature extraction [13,14]. Furthermore, DL’s ability to automatically learn high-level and meaningful features from high-dimensional data leads to high recognition rates, making it particularly suitable for learning complex patterns from data in intricate tasks like posture recognition.

Considering all this, there is a need for a holistic digital tool that facilitates ergonomists’ work and workers’ understanding of the posture risk, empowering them with a detailed, comprehensive, and understandable risk analysis. Hence, this manuscript aims to develop a framework that objectively assesses and quantifies the ergonomics of workers’ postures. It expeditiously provides information about the ergonomic risk the worker is exposed to and the impact of the postures’ accumulation. This information can be used by the workers, managers, and ergonomists to identify the risk source and possibly redesign tasks or re-educate postural habits, and, thus, prevent musculoskeletal disorders. The identification of the postures responsible for increasing the ergonomic risk takes advantage of DL models, due to their ability to recognise human movements continuously and automatically, without the need to manually define the movement features.

This study aims to answer the following research questions:

1. How can the accumulation of postural ergonomics risk over time be quantified?
2. How can ergonomic risk be associated with the specific postures that lead to it?
3. Can an ergonomic assessment tool be simultaneously easy to use and comprehensive for end users?

The main contributions of this work are:

1. The automation, using wearable sensors, of two ergonomic tools, one for agriculture and physically hazardous works.
2. The delineation of posture risk levels through an ergonomic index that takes into account the impact of previous postures on the actual ergonomic risk.
3. The identification of which postures lead to the highest ergonomic risk in the addressed activity sectors, based on the collected inertial data, by combining the posture recognition outputs with the computed ergonomic scores.
4. An easy-to-use graphical user interface (GUI) that presents an intuitive and comprehensive posture assessment, validated by ergonomists.

The paper is organised as follows: Section 2 explores how the previous literature studies implemented systems for posture assessment based on Inertial Measurement Units (IMUs); Section 3 proposes the overall framework for a holistic ergonomic assessment; Section 4 describes the methodology used to acquire and process the inertial data; Section 5 presents the results; Section 6 discusses them; and Section 7 summarises the main conclusions and prospects future work.

## 2. Literature Review

The existing methods for conducting ergonomic assessments are outlined in Section 2.1, whereas more focused research on wearable-based solutions for this purpose is presented in Section 2.2.

### 2.1. Ergonomic Risk Assessment Methods

In WRMSDs’ risk assessment, there are three main categories of methods for evaluating exposure to risk factors that lead to these injuries, which are presented in ascending precision and invasiveness as follows: self-reports, observational studies, and direct measurements. Self-reports collect data regarding exposure to physical and psychosocial factors and are based on interviews and questionnaires, in which workers are asked to estimate the prevalence of postures or the frequency of movements. Despite being straightforward to apply, their exclusive use may lack precision since workers’ perceptions are subjective and sometimes unreliable [3,15,16]. Hence, self-reports are frequently accompanied by other methods that provide more concrete data [16].

Observational methods depend on an observer and may be field-based or video-based, i.e., based on video recordings analysed offline by an expert. Field-based approaches are mostly used for static or repetitive jobs and typically rely on checklists [15,16]. Several observational assessment tools can be found, e.g., the Ovako Working Posture Analysing System (OWAS) [17], Postural Loading Upper Body Assessment (LUBA) [18], Rapid Upper Limb Assessment (RULA) [19], Rapid Entire Body Assessment (REBA) [20], Ergonomic Assessment Worksheet (EAWS) [21], NIOSH Lifting Equation [22], or Quick Exposure Check [23]. Despite being widely used, these methods also have the drawback of intra- and inter-observer variability [15]. In turn, video-based approaches are targeted to posture assessment in dynamic activities and empower more detailed evaluations since they include dedicated software to analyse the data objectively. Yet, these are not very convenient, they are time-consuming, require highly specialised staff, and their cost is higher than that of field-based methods [15].

Replacing observations with objective direct measurements, i.e., with sensors, can bring more accuracy and reproducibility to this analysis [2]. Motion Capture (MoCap) systems record movements and insert them in a 3D model of human kinematics [15,24]. Postures, defined by the body segments’ position and angular movement, can be tracked using marker-based methods, which attach optical, sonic, or electromagnetic markers to specific points of the body [15]. Optical MoCap systems, using either active (light-emitting LED) or passive (reflective) markers, like Vicon or Qualisys [24,25], are typically more accurate but are more suitable for laboratory-simulated scenarios. More recently, markerless technology, such as Microsoft Kinect, which integrates depth cameras and computer vision algorithms, has emerged in kinematic analysis. However, camera-based methods require constrained environments, highly depend on camera positions and light conditions, may suffer from occlusion [26,27] (which is likely to happen in dynamic tasks) and suffer from privacy issues since they record images [28]. In turn, inertial MoCap systems, such as Xsens or Synertial, are not affected by these problems [3,26]. Both vision-based methods and wearable inertial sensors are effective in ergonomic assessment tools; however, the best trade-off between accuracy and portability of the latter makes them very attractive for estimating body segments’ orientation and joint angles [29]. Nevertheless, these inertial MoCap systems, which are designed for generalised use (e.g., biomechanical analysis, rehabilitation, and game development), can still be quite expensive. Moreover, advances in wearable technology and the scientific community have been driving the development of more cost-effective wearable solutions based on IMUs [3,26]. For providing rigorous kinematic evaluations to manage and prevent WRMSDs and improving the knowledge of the underlying human motions [3], this study relies on wearable inertial MoCap systems.

### 2.2. Inertial-Based Posture Assessment Systems

Research has been conducted on the automation of ergonomic risk assessment methods using IMU-based systems. For instance, Ref. [30] applied RULA’s joint angle thresholds to IMUs’ and goniometers’ data in real time to perform the ergonomic assessment and provide visual and auditory feedback. Another study [26] complemented RULA in the sagittal plane with LUBA in the coronal plane and embedded the hardware in a smart vest capable of providing vibrotactile feedback. Both manuscripts revealed that biofeedback enabled an overall reduction of the time spent at high ergonomic risk levels, as well as increased posture self-awareness, suggesting that posture monitoring systems are a valuable tool for assisting the ergonomic analysis of hazardous work tasks.

However, these systems’ outcomes are not self-explanatory, as they do not indicate which types of posture conveyed the highest ergonomic risk. One study [31] also implemented a joint angle-based finite-state machine but combined the states to define overall postures. Nonetheless, this angle-based approach has limited complexity and generalisation. A hidden Markov model for posture recognition based on the EAWS method was implemented by [32], considering four taxonomy levels. However, this tool does not convey a measure of the risk. In [33], the authors carried out an ergonomic assessment by posture class using a hybrid model of a Convolutional Neural Network (CNN) with Long Short-Term Memory (LSTM), but no global score was provided, as postures were not classified based on body segments’ orientation.

Furthermore, none of these studies took into account the cumulative impact that previous postures have on the current ergonomic risk, as already pointed out in our previous literature review [34]. The kinematic wear model proposed by [35] tackled this, for a task allocation framework, by distinguishing wear and recovery phases, during which a joint kinematic wear index increases as a result of the accumulation of non-neutral postures and decreases with neutral ones, respectively, simulating the RC circuit charging–discharging.

Hence, there is a need to deconstruct the common simplistic posture assessments by breaking the risk into parts, i.e., into the different posture classes, so that the users can have a measure of how risky each posture is. It is also necessary to show workers the actual posture risk, which, unlike what current snapshot tools consider, is affected by previous postures.

## 3. Proposed System Overview

**Figure 1:**
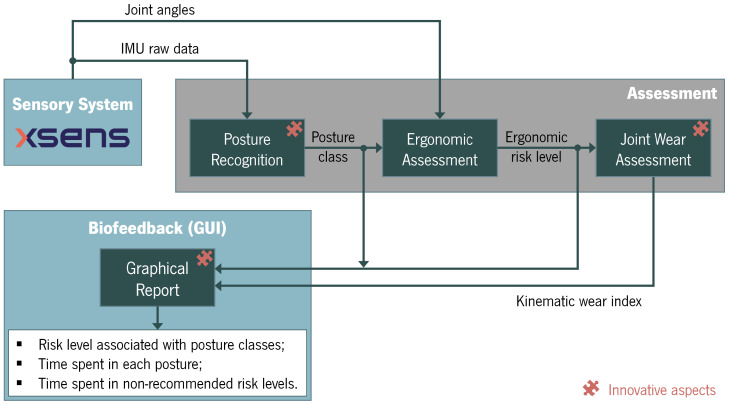
Conceptual design of the system.

The conceptual design of the proposed system for posture assessment is shown in Figure 1.

The inertial data are the primary input for the proposed system’s various modules, which are outlined below.

### 3.1. Posture Recognition

**Figure 2:**
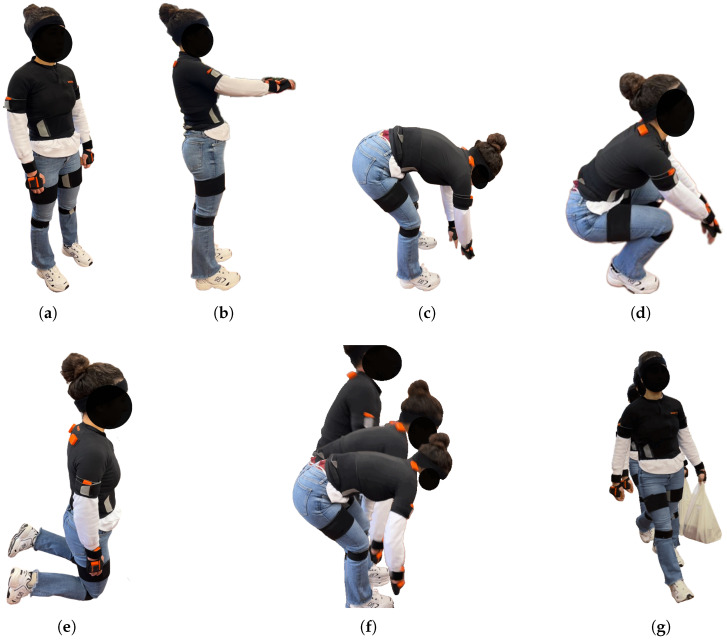
Identified typical postures to be recognised. (**a**) *Standing*; (**b**) *reaching*; (**c**) *stooping*; (**d**) *squatting*; (**e**) *kneeling*; (**f**) *lifting/lowering*; (**g**) *carrying*.

The posture recognition module aims to recognise the subject’s posture, among those identified as relevant to the addressed activities, using a neural network whose input is raw acceleration and angular velocity data. The postures considered relevant to recognise, represented in Figure 2, are those from our previous works [36,37] and are shared by both agriculture and construction workers as follows: *standing*, *reaching*, *stooping*, *squatting*, *kneeling*, *lifting/lowering*, *carrying*, and *others*.

Several DL architectures and hyperparameters were tested and studied in our previous work [37], using the dataset acquired by the team [38]. The model with the best test metrics (F1-score of 94.33% and accuracy of 95.29%, for an inference time of 0.29 ms per window), a hybrid CNN-Transformer with learnable fusion, was selected to be used in the present study. The architecture comprises two 2D and three 1D convolutional layers, 128 filters in the first layer, doubling with each layer, a kernel size of 5, eight encoder layers with 16 attention heads, and an embedding size of 128. The model uses 1 s windows without overlap as input and was trained with a learning rate of 10^−4^ and a batch size of 64 [37].

### 3.2. Ergonomic Assessment

The ergonomic assessment module aims to continuously quantify the user’s posture according to its deviation from the ideal neutral one, where all body segments are nearly aligned with gravity. Its inputs are the joint angles, and the outputs are the ergonomic risk levels, which reflect how far the user’s posture is from the ideal one. The assessed joints are the back, shoulders, and elbows, since the back and upper limbs are the most affected body parts by WRMSDs [1]. Two distinct ergonomic assessment methods were adapted as criteria for this assessment: Agricultural Whole-Body Assessment (AWBA) and LUBA. The users can select in the GUI which one to apply depending on their preference, since both consider different aspects and provide distinct insights about the user’s ergonomics, as will be detailed in Section 5.2.

The AWBA tool [39] combines Agricultural Upper-Limb Assessment (AULA) [40], which considers the three upper-body joints mentioned above to provide a risk level, and Agricultural Lower-Limb Assessment (ALLA) [41]. They were specifically developed for assessing commonly assumed postures in agricultural work (one of our use cases), concerning the sagittal plane (flexion/extension) alone. Their risk level classification was developed based on electromyography (EMG), heart rate, and self-reported discomfort. Both AULA and ALLA were demonstrated to be appropriate for estimating risky body postures that frequently occur in agricultural tasks, showing better agreement with expert evaluation than other evaluation tools, namely, REBA, RULA, and OWAS [40,41,42,43]. AWBA will also be used for the construction case, since both sectors share the most typical postures. As AULA and ALLA were primarily conceived as observational methods, these two tools provide loose angles instead of ranges; therefore, we partitioned the joint angles into ranges, using thresholds set at the values halfway between the angles provided. The lower-limbs’ ergonomic assessment requires the posture recognition module’s output, as ALLA distinguishes *kneeling* posture as a different category and assigns it a distinct risk level without resorting to the knee angle.

For the ergonomic analysis of postures, in this work, a finite-state machine was implemented to discretise the angles of the monitored joints (back, shoulders, elbows, and knees), acquired by the sensors, into a state (score/risk level). Table 1 and Table 2 indicate the values of the finite-state machine for the upper body and lower limbs, respectively, inspired by AULA and ALLA. Table 3 illustrates how the overall risk level is obtained.

In order to obtain a more specific analysis for the back, shoulders, and elbows, another ergonomic method was adopted as well. Furthermore, to provide a more complete assessment, it was decided to assess multiple back and shoulder motion types, namely, flexion/extension, lateral bending, and axial rotation for the back, and flexion/extension and abduction/adduction for the shoulders. Although RULA is the most employed ergonomic method, it oversimplifies the assessment for planes other than the sagittal. In turn, LUBA [18] appeared as the most suitable for this purpose, by providing angular thresholds for several upper-body joint motions, including the ones mentioned, and giving each an ergonomic score.

The finite-state machine for the LUBA-based ergonomic assessment is detailed in Table 4.

To provide a broader assessment that represents the overall ergonomic risk of the task, besides each joint separately, the entire upper-body posture was also quantified, with a global LUBA score that is the sum of each joint motion score when it is above 1 (the minimum score), as expressed by Equation (1), as follows:

(1) $$G=\sum_{i=1}^{n}\sum_{j=1}^{m_{i}}S_{ij}$$

where *G* is the global LUBA score, *i* is the joint (from Table 4), *j* the joint motion (also from Table 4), *n* the number of joints involved (5), $m_{j}$ the number of joint motions studied in the joint *i* (three motions for the back, two for each shoulder, and one for each elbow), and $S_{ij}$ the corresponding LUBA score. The global score takes into account only one of the arms, and, since both are used in the considered activities, in this study, the global assessment was performed using the arm with the highest sum of scores for the shoulder and elbow at each time step. Therefore, the applied scale varies between 0 and 62.

Following the LUBA guidelines, four posture categories regarding the need for corrective actions were defined, according to the calculated *G* score, as Table 5 clarifies.

### 3.3. Joint Wear Assessment

Despite the above-mentioned ergonomic scores providing an idea of the current risk, they were primarily conceived for a discrete analysis. Indeed, one of the shortcomings of typical ergonomic assessments is the disregard for the cumulative effect of previous ergonomic risks, as if the past had no impact on the true ergonomic risk at each time step. To address this, in this work, the kinematic wear index, introduced in [35] and inspired by the RC circuit-like behaviour of muscle fatigue, is used to enable a more complete and realistic assessment. Although this model neglects the effect of overloading forces, it remembers the time previously spent in hazardous postures as well as the repetitions. The kinematic wear index reflects not only the current postural risk but also the previous ones, accumulating all these ergonomic scores, for each joint and motion type, when the subject is in hazardous postures (wear phase)—representing the accumulation of stress in the joints—and decreasing when in a neutral posture (recovery phase). It varies between 0 (the ergonomically ideal value) and 1 (the worst). In the present work, different than [35], each type of joint motion is assessed—separately, since each joint motion is considered independent—and its kinematic wear increases when the associated ergonomic score in the current moment is above the minimum (wear phase), as expressed by Equation (2), as follows:

(2) $$V_{ij}\left(t\right)=1-\left(1-V_{ij}\left(t_{0}\right)\right)e^{-\int_{0}^{t}\frac{S_{ij}\left(q\left(\tau \right)\right)}{C_{ij}}\;d\tau }$$

where $V_{ij}\left(t\right)\in \left[0,1\right]$ represents the kinematic wear index of the joint *i* for the motion *j* (from Table 4) at instant *t*; $V_{ij}\left(t_{0}\right)$ is the kinematic wear index at the instant $t_{0}$, where the ergonomic score level ceased to be the minimum; q(τ) is the joint configuration; $S_{ij}\left(q\left(\tau \right)\right)$ is the ergonomic score associated with that angle, in this case, according to LUBA; and $C_{ij}$ is the endurance capacity for that joint motion. On the other hand, when the joint is at the minimum risk level, it is recovering, hence, the kinematic wear index, $V_{ij}$, decreases as defined in Equation (3), as follows:

(3) $$V_{ij}\left(t\right)=V_{ij}\left(t_{0}\right)e^{-\frac{r_{ij}}{C_{ij}}t}$$

where $V_{ij}\left(t_{0}\right)$ is the kinematic wear index at the instant $t_{0}$, where the ergonomic score became the minimum; and $r_{ij}$ is the recovery rate associated with that joint motion.

The endurance capacity, $C_{ij}$, is given by inverting Equation (2), considering $V_{ij}\left(t_{0}\right)$ = 0, $V_{ij}\left(t\right)$ = $V_{max}$ = 0.993 (which corresponds to the kinematic wear at the time step of five time constants, when a capacitor in an RC circuit is typically considered to be fully charged); the average LUBA score $S_{ij,avg}$ of the motion *j* of joint *i* (calculated between the minimum and the maximum of the scale); and a $T_{max}$ = 240 s (corresponding to the time until a subject applying a low force feels physical discomfort, in a static configuration, as stated by [19]). Differently from [35], in our work, the endurance capacity was defined for each joint motion individually, as follows:

(4) $$C_{ij}=-S_{ij,avg}\frac{T_{max}}{\ln\left(1-V_{max}\right)}$$

Regarding the recovery rate, $r_{ij}$, which is also specific for each joint motion, Equation (5) is obtained by inverting Equation (3) in order to match the recovery time with the wear time, i.e., a decrease of five time constants, starting at $V_{ij}\left(t_{0}\right)=V_{max}$, as follows:

(5) $$r_{ij}=-\frac{C_{ij}}{T_{max}}\ln\left(\frac{1-V_{max}}{V_{max}}\right)$$

**Figure 3:**
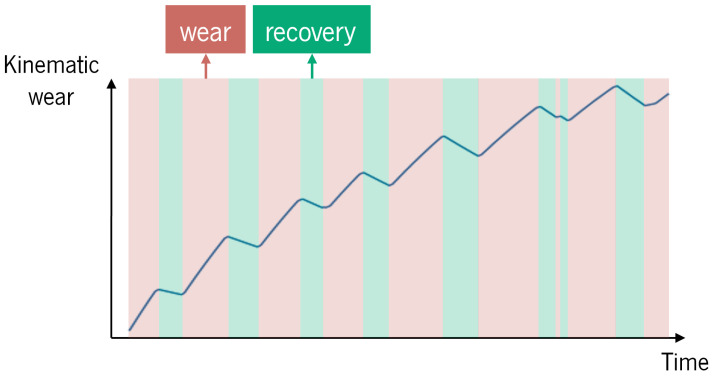
Behaviour of the joint kinematic wear index, $V_{ij}$, calculated using Equation (2) during the wear phase and using Equation (3) during the recovery phase.

Figure 3 shows how the index changes over time, during the wear and recovery phases, respectively.

With this index, new risk levels considering the cumulative effects of postural risk can be defined. In this manuscript, two levels were considered, using a kinematic wear index threshold of 0.7, as established by [44] for a muscle fatigue model. For values below that, the risk is considered minimal, and the subject does not need to change posture yet. For values of kinematic wear index above 0.7, the risk is high, corresponding to a high accumulation of non-recommended postures, which means that the subject should change to a neutral posture. The percentage of time above the threshold, which reflects how long the accumulated posture hazard exceeded the recommendation, was calculated. This kinematic wear model could also be integrated into a real-time biofeedback strategy, in which the user surpassing the aforementioned threshold would trigger biofeedback cues, namely, haptic cues.

### 3.4. Report for Biofeedback

The solutions presented by the framework ErgoReport for an automated and complete posture assessment were conceived to meet the ergonomists’ needs. Hence, an unstructured interview with two ergonomists helped in understanding the main issues they face when conducting a postural risk assessment, which are listed in Table 6. The recognised postures and the computed ergonomic metrics enabled the generation of this report, in which the ergonomic score is associated with the postures that generated it. This is, to the best of our knowledge, the first approach to, simultaneously, automatically identify the type of posture being performed with DL and assign a quantitative score to the user’s ergonomics. The computed information is presented through several representations in the ErgoReport, as follows:

- The visualisation over time of the ergonomic risk as well as the kinematic wear index for each joint (only using LUBA), to allow us to understand how each ergonomic score contributes to joint stress.
- The visualisation over time in a single plot of the sequences of ergonomic scores and performed postures. This acts like a playback of the worker’s shift and can be particularly useful when the ergonomic scores are very high within specific time intervals, allowing for a detailed examination of the sequence of postures over time.
- A summary of the work shift presenting the time for which each posture was held and the average ergonomic score associated with each posture class. This enables a straightforward visualisation of the most hazardous posture classes, which require attention. It is relevant to show these two aspects jointly, as certain postures may have a lower average risk than others but may represent a larger time percentage. The report also mentions the joint motions that surpassed the kinematic wear threshold of 0.7 for more time. By computing these ergonomic metrics for subjects individually and for all subjects together, the managers become capable of assessing whether the task itself is ergonomically inappropriate or if the problem lies in the postures of certain workers.

## 4. Materials and Methods

Data acquisition was conducted inside a laboratory in the School of Engineering of the University of Minho, in alignment with the ethical procedures of the Ethics Committee in Life and Health Sciences (CEICVS 147/2021), following the standard set by the declaration of Helsinki and the Oviedo Convention.

### 4.1. Participants

A total of 13 subjects (9 males and 4 females; age: 24.3 ± 1.9 years old) from the academic community of the University of Minho accepted to participate in the data acquisition. Very physically distinct participants (body mass: 67.0 ± 11.7 kg; body height: 172.2 ± 11.4 cm; hip height: 95.8 ± 8.0 cm; arm span: 170.4 ± 12.6 cm) were selected to obtain a dataset that considers the possible variability associated with the physical stature in posture execution. Also, the subjects were selected based on the following inclusion criteria: healthy, without clinical history or evidence of motor injuries.

### 4.2. Hardware Setup

The participants were instrumented with two full-body inertial MoCap systems from Xsens MTw Awinda (Movella Inc., Henderson, NV, USA), a state-of-the-art system. The first one was connected to software Xsens MT Manager 2022.0 (Movella Inc., Henderson, NV, USA), which provided the input for the posture recognition module (raw acceleration and angular velocity). The second system was connected to proprietary software Xsens MVN Analyze 2021.0 (Movella Inc., Henderson, NV, USA) to provide the joint angles to the ergonomic assessment module. Only seven body locations—sternum, right shoulder, right forearm, left forearm, left upper leg, right lower leg, and left foot—were selected, as these were demonstrated to be the most statistically relevant in our previous study [37]. The two sets of IMUs were stacked with tape. The second one was placed on the bottom, closer to the body. The sensors were placed, following the manufacturer’s guidelines (https://base.xsens.com/s/article/Sensor-Placement-in-Xsens-Awinda-System, accessed on 13 March 2023), over bony landmarks and tightened with straps. The sampling frequency was set to 20 Hz, respecting the Nyquist theorem, since, in our previous work [37], we verified that the signals’ frequencies in the spectrum lay below 5 Hz. The literature also reports the human movement frequencies to be mostly between 0.3 and 3.5 Hz, with a maximum of 10 Hz [45]. Moreover, 20 Hz was shown to be optimal for activity recognition [46].

### 4.3. Protocol

After the participants’ instrumentation, their anthropometric data were collected to adjust the biomechanical model from Xsens MVN Analyze software for them. The ground truth sensory system was calibrated in N-pose (neutral posture), performing the necessary repetitions until achieving good calibration quality. The other system’s IMUs were turned on after that, to avoid interference.

The participants were instructed to perform two task circuits that replicated real worksites in the agriculture and construction sectors. The protocol was explained without imposing a way to perform each posture. Moreover, participants were told to carry out the tasks at their own pace, since they were not actual workers, and fixed time constraints could induce mental stress.

**Figure 4:**
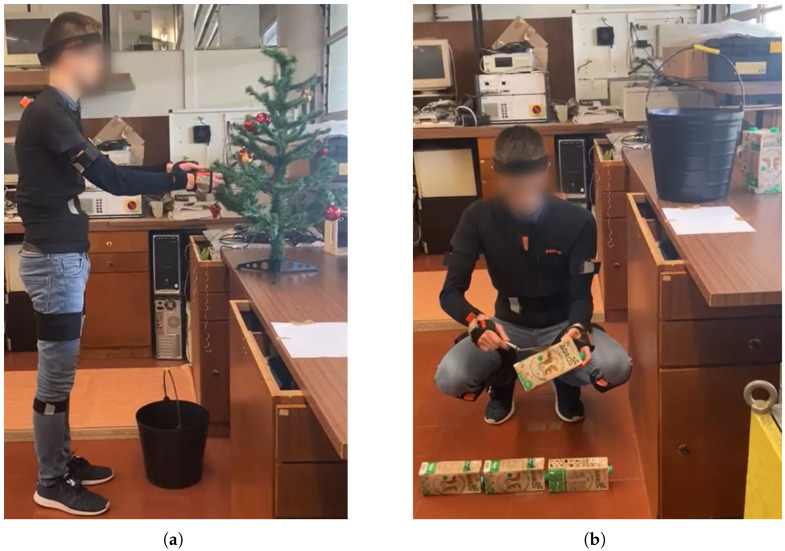
Experimental setup for the (**a**) harvesting (agriculture) and (**b**) bricklaying (construction) tasks.

One of the tasks replicated an agriculture task—harvesting and transporting crops. The subjects were to pick a total of 12 Christmas baubles (simulating fruits) from an artificial tree at a height of 90 to 180 cm and put them into a bucket on the floor, one by one, at three sites during each trial, transporting the bucket between each of the harvest sites, located 2.5 m apart. A frame of one trial is presented in Figure 4a.

A construction task circuit was also performed. Mimicking bricklaying, it consisted of carrying a bucket with six milk cartons (simulating bricks) for 4.5 m, from a starting point to the work site, located one subject-foot from a 90-cm-high workbench, on top of which the subject should place a bucket, and a sheet of paper acted as a mortar tray. The subjects had to stack the milk cartons in two vertical layers to simulate the construction of a wall. With a trowel, the subject simulated spreading mortar on the floor before starting a new layer and on the sides of each milk carton, as shown in Figure 4b.

### 4.4. Framework Implementation

The whole pipeline was implemented using Pytorch and Pytorch Lightning libraries in a Python environment. We used a personal laptop with the following specifications: GPU: 1× Nvidia GeForce GTX 1050; CPU: Intel(R) Core(TM) i7-7700HQ @ 2.80GHz (4 core, 8 threads); RAM: 16 GB.

**Figure A1:**
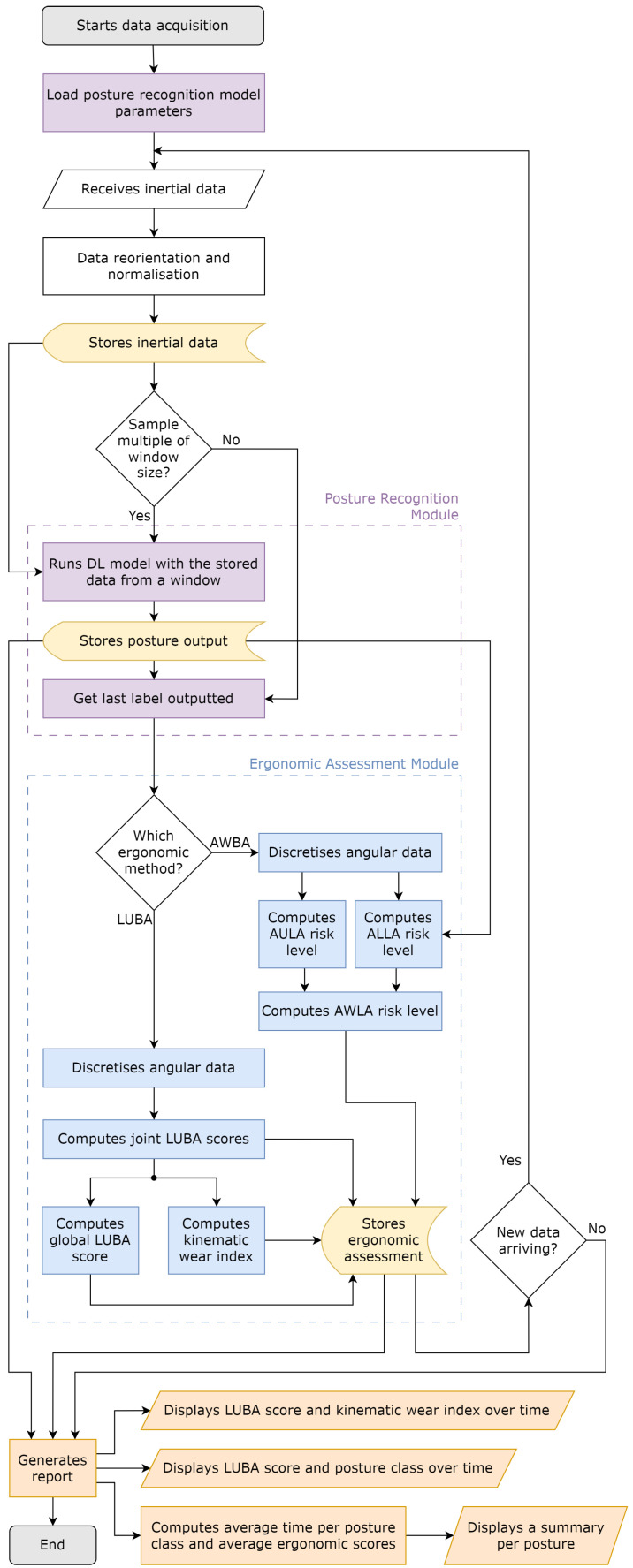
Flowchart of the proposed holistic ergonomic assessment framework.

The flowchart of the proposed framework is depicted in Figure A1 (Appendix A). Although the proposal is to receive the data one sample at a time (real-time) to allow the future integration of haptic biofeedback cues, which is within the scope of this manuscript, the modules were executed offline, and the data were loaded and processed all together. Note that, with the LUBA ergonomic assessment, the posture class is only needed for generating the report, while, with AWBA, it is also needed for calculating the risk. Both the posture recognition and the ergonomic assessment modules use the data at 20 Hz, but, while the former treats the data in time windows 1 s in size (no overlap), the ergonomic assessment metrics are calculated for each sample.

**Figure A2:**
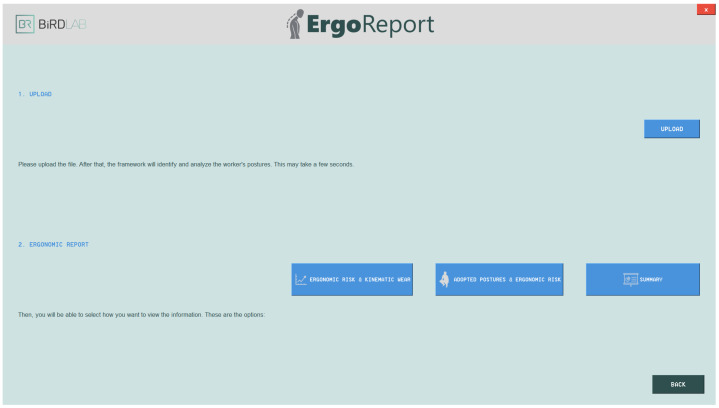
GUI of the developed framework, showing the page with the options to upload the acquired data and perform the posture assessment.

Figure A2 (in Appendix A) shows one of the pages of the developed framework’s GUI, where the user can upload the data acquired by the wearable sensors and select the type of analysis from the ones mentioned in Section 3.4.

### 4.5. Data Preprocessing

The collected data underwent the same preprocessing steps for the DL model development, as described in [37].

The raw acceleration and angular velocity were reoriented so that all sensors share the same orientation, with all axes aligned with a reference frame. The original orientation of each IMU was assumed to be the same for all subjects. This is considered reasonable since their instrumentation was verified at the beginning of each trial, and whenever a slippage occurred, the trial was stopped, discarded, and repeated.

To minimise bias in the neural network for posture recognition, as different sensors may have distinct ranges and sensitivities [47], MinMax normalisation between 0 and 1 was applied to the data. The data were normalised feature-wise using the extreme values of the training set, as the DL model was also trained with data normalised within this range.

### 4.6. Report Usability Assessment

To assess ErgoReport’s usefulness and usability, ergonomists were invited for an online interview where we presented the system’s goal and the use cases. A demonstration of how to use the GUI, with some example trials, was conducted.

At the end, the ergonomists were asked to fill out a 7-point Likert scale questionnaire (1: strongly disagree; 7: strongly agree). This custom questionnaire addresses three main aspects of the report—its usefulness, ease of use, and usability—and was adapted from the following three different questionnaires: Perceived Usefulness and Ease of Use (PUEU) [48], Interface Usability Instrument (INUIT) [49], and Software Usability Measurement Inventory (SUMI) [50]. With this custom questionnaire, new specific directions for improvement can be pointed out by ergonomists, who are end users.

## 5. Results

### 5.1. Joint Kinematic Wear

**Figure 5:**
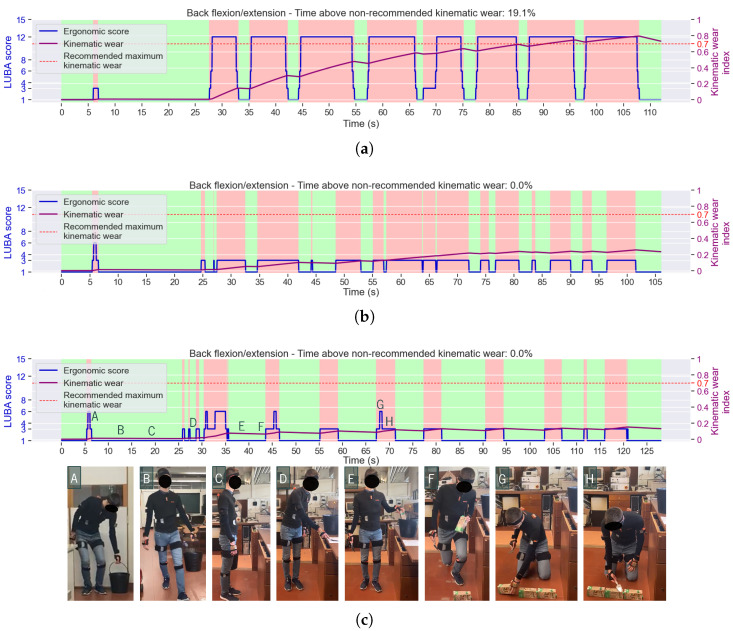
Back flexion/extension LUBA scores and kinematic wear during the circuit based on the construction task, adopting (**a**) *stooping*, (**b**) *squatting*, or (**c**) *kneeling* postures for bricklaying. Red and green stripes highlight, respectively, the wear and recovery phases. The red dashed line indicates the kinematic wear threshold of 0.7, suggested by [44]. To illustrate the trials, some frames (A to H) from the latter example’s recording are presented and identified in the plot.

The kinematic wear index was computed for nine joint motions. Figure 5 presents one of the ErgoReport’s graphs, with the kinematic wear of one of the most concerning joint motions, the back flexion/extension. Three different cases are exposed, referring to three trials of construction tasks from the same subject, one using *stooping* posture for bricklaying (Figure 5a), and the others using *squatting* (Figure 5b) and *kneeling* (Figure 5c), starting with zero as the initial conditions. These figures reflect the temporal dynamics of the tasks performed. In the three cases, as represented in frame B in Figure 5c, the *carrying* of the bucket took place until t≈ 20 s, when the subject arrived at the bench (frame C). This did not require any back flexion; thus, the back kinematic wear index did not increase much. It only increased when grabbing the bucket from the ground in the beginning, shown in frame A. Still, this index remained negligible until t≈ 25 s. After that, risky postures started to be frequently adopted, first when the subject reached the mortar in the tray (frame D), and, then, when he bent to the floor to spread it, achieving ergonomic scores above the minimum in those instants, which translated into an increase in the back kinematic wear index. At t≈ 35 s in the trial of Figure 5c, when the subject got up to reach a milk carton (frame E), he straightened his back, which led the LUBA score to reach the minimum (1) and, consequently, induced a decrease (recovery) in the back kinematic wear regarding flexion/extension motion. Then, the participant lowered his body (frame F) for the *kneeling* motion, to spread the mortar on the milk cartons and lay them on the “wall” (as shown in frames G and H). It required considerable back flexion, which, for *kneeling*, typically leads to a LUBA score of 3 or 6. With this, the joint kinematic wear escalated every time the subject had to place the simulated brick on the “wall” and decreased every time the participant got up to pick another carton—during this time, the subject’s back was able to recover.

Note that, in Figure 5a, during the last fifth (after *t* = 90 s) of the trial where *stooping* was used for bricklaying, the back kinematic wear was above the defined threshold of 0.7 for the recommended maximum accumulation of joint stress. Other joints also surpassed that value, for instance, the shoulders, regarding flexion/extension. This resulted from the accumulation of joint stress when the subjects were in the *stooping* posture, where the shoulder flexion angle was above 90^∘^. Accordingly, back and shoulder flexion/extension are the joint motions that should require more attention from the workers.

### 5.2. Association Between Ergonomic Risk and Postures

**Figure 6:**
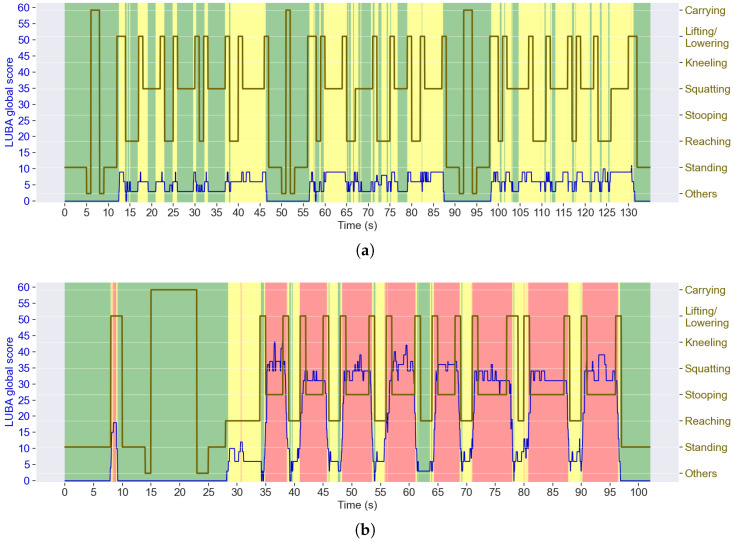
Global LUBA scores (on a scale from 0 to 62) and postures adopted during (**a**) an agriculture and (**b**) a construction trial. The background colours refer to the categories regarding the need for corrective actions, defined in Table 5.

The report associates the ergonomic assessment with the postures recognised by the framework. In a continuous way over time, Figure 6 shows that the highest LUBA scores occurred in the moments of *stooping*. Figure 6b shows, e.g., LUBA score peaks during *stooping* repetitions, which correspond to the act of placing the milk cartons on the floor and spreading mortar on them, where the subjects have to bend the back, almost always corresponding to the most concerning LUBA category (IV) and achieving a maximum global score of 43 out of 62 for that subject specifically. In comparison, *squatting* postures in Figure 6a were associated more often with category II (global score between 5 and 10).

**Figure 7:**
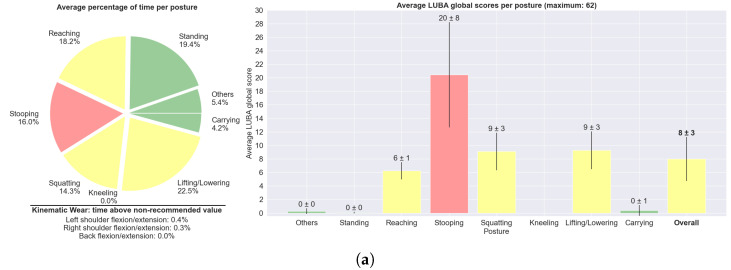

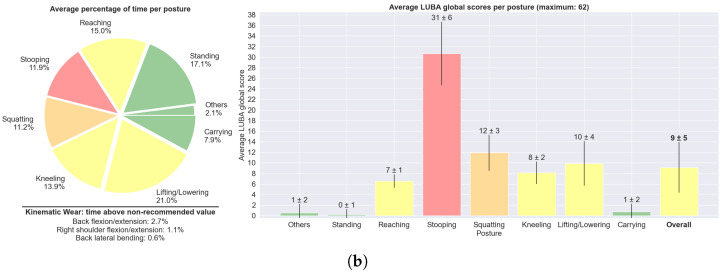
Summary report with the average percentage of time spent at each posture, averages of the mean LUBA scores (on a scale from 0 to 62) for each posture and the 3 joint motions that breached the kinematic wear threshold of 0.7 for the longest time, considering all (**a**) agriculture and (**b**) construction trials. Note that the time percentages do not have to add up to 100%, as these are the averages of all trials. The colours refer to the categories regarding the need for corrective actions, defined in Table 5.

Figure 7 displays an overview of the tasks carried out, encompassing all trials from all subjects, for the agriculture and construction task circuits separately. It transmits information regarding the average percentage of time spent at each posture, as well as the average ergonomic score (here, the global LUBA score) for each posture, and the three joint motions that breached the kinematic wear threshold for more time. *Lifting/lowering* was the posture that was held for a longer period of time, since the protocol, primarily designed to train the DL models, included a huge number of transitions. Both harvesting and bricklaying tasks were associated with a considerable global ergonomic score. For harvesting, the overall score was 8, which, according to LUBA guidelines, would mean no need for immediate corrective actions. For bricklaying, the overall score was 10, meaning there is a need for corrective actions soon. Only *standing* (neutral posture), *carrying*, and *others* were free of risk. *Stooping* was the posture with the highest risk score in both task circuits.

**Figure 8:**
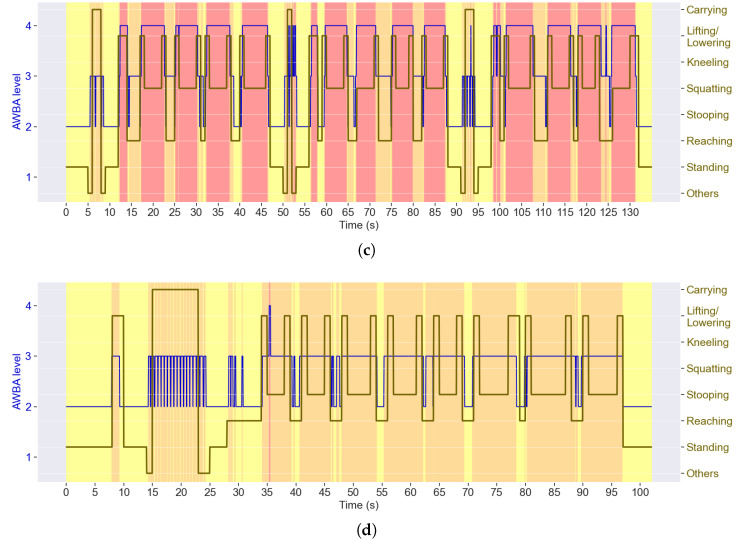
AWBA risk levels (on a scale from 1 to 4) and postures adopted during (**a**) an agriculture and (**b**) a construction trial.

Using the ergonomic method AWBA instead, several differences become noticeable in Figure 8, which presents the ergonomic assessment for the same trials from Figure 6. With AWBA, *squatting* posture is associated with a higher risk than *stooping*, contrary to what was verified with LUBA.

**Figure 9:**
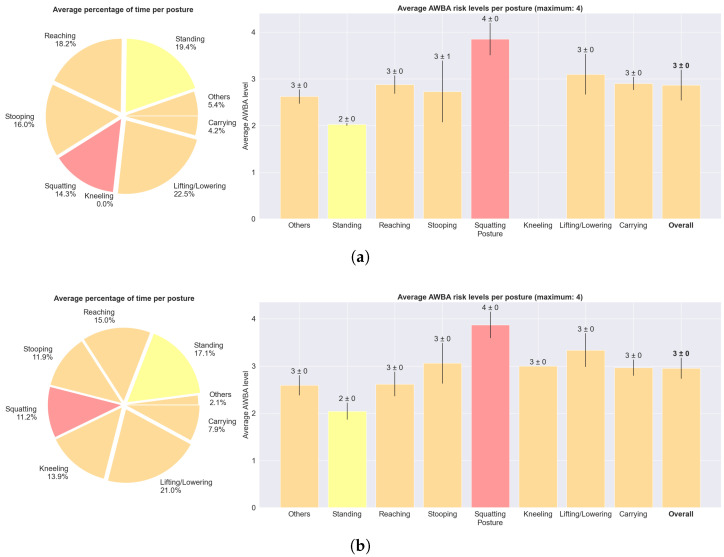
Summary report with the average percentage of time spent at each posture and averages of the mean AWBA risk levels (on a scale from 1 to 4) for each posture, considering all (**a**) agriculture and (**b**) construction trials. Note that the time percentages do not have to add up to 100%, as these are the averages of all trials. The colours refer to the risk levels, as defined in Table 1.

Figure 9 presents the summary report considering the AWBA-based ergonomic assessment. By comparing with Figure 7, it can be seen that AWBA generally rates subjects’ postures as riskier than LUBA, since ALLA assigns the risk level 1 only to sitting postures, which were not addressed in this study. Only *standing* was considered to have a risk below 3. In agreement with Figure 8, the *squatting* posture was assigned the highest average risk.

### 5.3. Report Usability and Usefulness

Seven ergonomists were invited to participate in the report usability study. Of these, five agreed to participate. However, one was subsequently unable to participate, and another one was interviewed but was not able to further fill out the questionnaire. Hence, four ergonomists participated in the study but only three completed the questionnaire.

According to the results of the custom questionnaire, in Table 7, the ergonomists agreed that the postural ergonomic report would be useful (Q6) in increasing their productivity (Q1, Q3, and Q5) and performance (Q2, Q4) and it would be easy to use (Q7–Q9). They found the desired information (Q15) and were able to easily understand it (Q10), although some suggested that other options could be added, such as the display of the joint angles over time. Some of them considered that the presentation of the information and the layout were a little confusing (Q11 and Q12).

## 6. Discussion

### 6.1. How Can the Accumulation of Postural Ergonomics Risk over Time Be Quantified?

The joint kinematic wear index allowed representing the accumulation of the ergonomic scores. This model distinguishes wear phases, where joint stress accumulates due to the ergonomic score above the ideal, from recovery phases, where the joint is not at risk. Otherwise, the LUBA ergonomic score, as represented in Figure 6 and Figure 7, does not consider the impact of maintaining hazardous postures over time (nor the AWBA risk). However, as can be seen in Figure 7 by the time spent above the joint kinematic wear index threshold of 0.7, in about only 2 min, the back and shoulder joints in the flexion/extension motions accumulated a lot of stress and were not far from that index maximum (on average), with the back spending 3.3% of the time above that limit for construction task circuits.

The joint kinematic wear model also discerns the impact of each ergonomic risk level. For instance, the trial where *stooping* was the posture chosen for bricklaying was the one that required the greatest back flexion, and, consequently, higher scores (12) were reached, according to LUBA. This translated into higher values of the back kinematic wear for the flexion/extension motion (Figure 5a). It can be seen that, in the time intervals where the LUBA score was 12, the index slope was steeper, meaning that the accumulation of stress in the joint was faster than when the LUBA score was 6 or 3—this difference is visible at *t* = 70 s in Figure 5a. This is corroborated by Equation (2), which indicates that the riskier the joint angle values, the faster the kinematic wear increases. Regarding recovery (minimum LUBA score), it can be seen in any of the figures that the higher the initial kinematic wear (i.e., the more accumulated posture hazard), the steeper the index descent, which is supported by Equation (3). Particularly, it can be seen in Figure 5a that the recovery that begins at *t* = 108 s with a kinematic wear index equal to 0.8 is much faster than the one that begins at *t* = 33 s with the index below 0.2.

These results point out the suitability of this model to describe joint usage over time. Still, although this model is inspired by the charge–discharge behaviour of muscle fatigue, the proposed approach has limitations concerning the provided information and its theoretical validation, as stated by [35]. For instance, considering the trials’ short duration (about 2 min) compared with a typical 8-hour work shift, the rapid exceeding of the threshold suggests that the chosen value—following [44]—may not be suitable. Furthermore, this threshold could be adapted according to the posture being performed, as some work tasks may require postures that entail a higher ergonomic risk. Hence, further investigation is needed.

### 6.2. How Can Ergonomic Risk Be Associated with the Specific Postures That Lead to It?

ErgoReport presented an association between ergonomic scores and postures. It showed, e.g., that *stooping* was the posture class with the highest risk score in both task circuits. This confirms what Figure 5 and Figure 6 have also indicated— *stooping*’s great back flexion is frequently associated with the highest score for that joint motion. In turn, the equivalent postures of *squatting* and *kneeling* were associated with much lower scores, especially the *kneeling* (performed only for the construction task circuit), suggesting that these could be a safer alternative to *stooping* for the same tasks.

Regarding the differences in the ergonomic scores between LUBA and AWBA, for instance, the highest score for *squatting* with the latter method, the reason is the consideration of the legs in the AWBA-based ergonomic assessment (Table 2). Although *stooping* is typically associated with a more prominent back angle than *squatting*, the knee angle is ergonomically less risky for *stooping*, being usually at risk level 2. Although this may sound odd, the truth is that most subjects perceived *stooping* as less demanding; thus, AWBA reflects better subject perception, which makes sense as it was conceived under similar considerations [39]. However, AWBA does not seem to be suitable for dynamic tasks, as evinced by the risk levels during *carrying*, where the ALLA level for the legs is constantly changing while the subject walks, leading to an oscillating pattern. Also, the minimal risk level assigned within the scope of this study was 2, as the AWBA considers that level 1 is only assigned to sitting postures, which were not present in the experimental protocol. Thus, modifications to this ergonomic method would be beneficial.

### 6.3. Can an Ergonomic Assessment Tool Be Simultaneously Easy to Use and Comprehensive for End Users?

The proposed ergonomic tool was assessed by ergonomists. Regarding usefulness, they recognised that this framework would expedite their work and allow them to obtain detailed and objective data for the posture assessment. The interviewed ergonomists considered that the interface would be easy to use after a quick explanation of its functioning. Concerning usability, there is still room for improvement. Some of the ergonomists considered that visualising the ergonomic risk and the joint kinematic wear index over time in the same graph (Figure 5) was slightly confusing at the beginning, although it became more straightforward after the authors’ explanation. Similarly, two ergonomists considered that the visualisation of the sequence of postures and their ergonomic risks over time (Figure 6 and Figure 8) was a little confusing, suggesting that it would be more intuitive if the posture class was displayed only when hovering the mouse over a certain time step. On the other hand, they considered that the graphs displayed in Figure 7 and Figure 9 are an important feature of this tool for providing a straightforward summary of the ergonomic performance, which they can be further investigated by visualising the graphs with the assessment over time. In fact, typical difficulties experienced by ergonomists are realising the sequences of postures performed by the workers, the time each one was held and which ones lead to the highest ergonomic risks throughout the shift, as we collected in our first survey (Table 6). Moreover, they considered that the colours expedited the association with risk levels. An ergonomist pointed out that the designations of the posture classes in the graphs could be replaced by representative images to expedite the association with each one.

## 7. Conclusions and Future Work

Motivated by the enormous impact of WRMSDs, this manuscript addressed one of their most relevant causes—hazardous postures. Focusing on the two activity sectors with the highest WRMSDs’ prevalence, we developed a valuable tool for providing a more targeted analysis of the underlying causes of high ergonomic risk and demonstrating the applicability of DL-based posture recognition to complement a postural ergonomic assessment. With ergonomists’ validation, within this framework, two ergonomic methods were automated based on inertial data as follows: the agriculture-specific AWBA, to assess the back, shoulders, elbows, and knees in the sagittal plane; and the standard ergonomic method LUBA, to assess the upper-body joints in the sagittal, coronal, and axial planes. In order to take into account previous postures’ impact on joint stress, a kinematic wear index was computed for the various joint motions. With regard to this, two situations were distinguished as follows: when the LUBA score is not the lowest, the joint kinematic wear index increases—and its rate of increase is greater when the risk is greater—representing the accumulation of posture hazards over time; contrariwise, for the minimum global score, the joint recovers and the kinematic wear index decreases. In a GUI, this index was plotted over time to show its evolution, and for each time step, the computed ergonomic score was associated with the posture class (recognised by the DL model), also enabling the assessment of the average risk of each posture and the overall risk of the entire tasks, with graphs. With these data, the user can understand which posture classes are the most hazardous. This GUI was evaluated by ergonomists, who considered it useful in expediting their work and easy to use. Concurrently, they provided relevant feedback towards an effective application of such a tool.

However, this study also has some limitations that need to be addressed in the future. The presented system was validated with volunteers simulating agriculture and construction activities, but an experimental protocol with workers in their real workplaces should be carried out. The kinematic wear index inspired by muscle fatigue behaviour should be validated with EMG, by associating the monitored joints with the corresponding muscles. A user-specific risk assessment is intended to be performed, considering the psychosocial and individual factors which most influence WRMSDs’ development. The drafted biofeedback strategy should be implemented in real time with vibrotactile cues, which could be triggered considering the accumulated joint stress, by using a kinematic wear threshold. Regarding the visual report, ergonomists’ suggestions to improve comprehensibility will be followed. The commercial MoCap system used is intended to be replaced by the team’s low-cost upper-body smart garment, Ergowear [51], implementing sensor fusion algorithms to estimate the joint angles.

## Figures and Tables

**Table 1:** Finite-state machine for the ergonomic assessment based on the AULA method.

Joint Flexion/Extension Angle Range (°)			
**Back**	**Shoulder**	**Elbow**	**Level**
α≤ 22.5	β≤ 22.5	Any	1
	22.5 <β≤ 72.5		2
	72,5 <β≤ 105		3
	β> 105		4
22.5 <α≤ 72.5	β≤ 72.5	γ≤ 22.5	2
		γ> 22.5	3
	β> 72.5	Any	3
α> 72.5	Any		2

Note: For clarity, the colours green, yellow, orange, and red are associated with each risk level, from 1 to 4, in ascending order.

**Table 2:** Finite-state machine for the ergonomic assessment based on the ALLA method.

Posture	Knee Flexion/Extension Angle Range (°)	Level
Standing/squatting	δ≤ 15	2
	15 <δ≤ 45	3
	45 <δ≤ 135	4
	δ> 135	3
Kneeling	Any	3

Note: Risk level 1 is not present, as ALLA assigns it only to sitting postures, which were not within the scope of this work.

**Table 3:** AWBA rules.

		**AULA Level**			
		1	2	3	4
**ALLA** **level**	1	1	2	3	3
	2	2	2	3	4
	3	3	3	3	4
	4	3	4	4	4

**Table 4:** Finite-state machine for the ergonomic assessment based on the LUBA method.

Joint	Joint Motion	Angle Range (°)	Score
Back	Flexion (+)/extension (−)	$\alpha_{fe}\leq$−30	15
		−30 $<\alpha_{fe}\leq$ -20	8
		−20 $<\alpha_{fe}\leq$ -10	4
		−10 $<\alpha_{fe}\leq$ 30	1
		30 $<\alpha_{fe}\leq$ 60	3
		60 $<\alpha_{fe}\leq$ 90	6
		$\alpha_{fe}>$ 90	12
	Lateral bending	$|\alpha_{lb}|\leq$ 10	1
		10 $<|\alpha_{lb}|\leq$ 20	4
		20 $<|\alpha_{lb}|\leq$ 30	9
		$|\alpha_{lb}|>$ 30	13
	Axial rotation	$|\alpha_{ar}|\leq$ 20	1
		20 $<|\alpha_{ar}|\leq$ 60	3
		$|\alpha_{ar}|>$ 60	10
Shoulder	Flexion (+)/extension (−)	$\beta_{fe}\leq$−60	10
		−60 $<\beta_{fe}\leq$−45	6
		−45 $<\beta_{fe}\leq$−20	3
		−20 $<\beta_{fe}\leq$ 45	1
		45 $<\beta_{fe}\leq$ 90	3
		90 $<\beta_{fe}\leq$ 150	6
		$\beta_{fe}>$ 150	11
	Abduction (+)/adduction (−)	$\beta_{aa}\leq$−30	8
		−30 $<\beta_{aa}\leq$−10	2
		−10 $<\beta_{aa}\leq$ 30	1
		30 $<\beta_{aa}\leq$ 90	3
		$\beta_{aa}>$ 90	7
Elbow	Flexion (+)/extension (−)	$\gamma_{fe}\leq$ 45	1
		45 $<\gamma_{fe}\leq$ 120	3
		$\gamma_{fe}>$ 120	5

**Table 5:** Posture categories regarding the need for corrective actions defined by the LUBA method.

Category	Global Score	Corrective Actions
I	G≤ 5	Not needed
II	5 <G≤ 10	In the next regular check (immediate intervention not needed)
III	10 <G≤ 15	Redesigning workplaces or working methods soon
IV	G> 15	Immediate

Note: For clarity, the colours green, yellow, orange, and red are associated with each category, from I to IV, in ascending order.

**Table 6:** Typical ergonomists’ difficulties in postural risk assessment and the corresponding ErgoReport’s solution to address it.

Ergonomists’ Difficulty	Framework’s Solution
Visualisation of the ergonomic risk along the work shift	Plot with the evolution of the LUBA scores of the various joint motions and the corresponding kinematic wear index over time, using colours to highlight when the accumulation of joint stress is below (green) or above (red) the recommended maximum
Realising the sequences of postures performed by the workers throughout the shift	Plot with the sequences of the ergonomic scores and the performed postures over time, coloured with green, yellow, orange, and red, for each of the LUBA posture categories to indicate corrective actions, facilitating the interpretation of the ergonomic risk; similar colours were assigned to each of the AWBA scores
Realising which postures led to the highest ergonomic risks along the work shift	
Realising which postures led to the highest ergonomic risks overall	Graph of the average ergonomic score associated with each posture class; and indication of the joint motions that surpassed the recommended maximum kinematic wear for longer cumulative durations
Knowing the time each posture was held	Pie chart with the time per posture—on average, when considering multiple subjects or multiple recordings from the same subjects

**Table 7:** Results of the 7-point Likert scale questionnaire for ErgoReport’s usability assessment.

Adapted from	Category	Question		Mean	STD
PUEU [48]	Usefulness	1.	Using the system in my job would enable me to perform the postural ergonomic assessment more quickly.	6.00	0.82
		2.	Using the system would improve my job performance, by providing more information and types of postural ergonomic assessment.	6.33	0.94
		3.	Using the system in my job would increase my productivity, since it automates the postural ergonomic assessment.	5.67	0.94
		4.	Using the system would enhance my effectiveness on the job, by providing more information about the workers’ postures.	6.00	0.82
		5.	Using the system would make it easier to do my job.	5.67	1.25
		6.	I would find the system useful in my job.	6.33	0.47
	Ease of use	7.	Learning to perform the postural ergonomic assessment with the system would be easy for me.	5.33	1.25
		8.	It would be easy for me to become skilful at using the system.	5.67	0.94
		9.	I would find the system easy to use	5.67	0.94
INUIT [49]	Usability	10.	I could easily understand the provided information.	5.67	0.94
		11.	I was not confused using the interface/graphical information.	4.67	0.47
		12.	I was not distracted by elements of the interface/graphical information.	4.00	0.82
		13.	Typography and layout added to readability.	5.00	0.82
		14.	The information presented was adequate for the space in the interface.	5.67	0.47
		15.	The information I desired was easily reachable.	5.67	0.94
SUMI [50]		16.	I would recommend this software to my colleagues.	6.00	0.82
		17.	I would like to use this software every day.	5.67	0.94

STD: standard deviation.

## Data Availability

The raw data supporting the conclusions of this article will be made available by the authors upon request.

## Abbreviations

The following abbreviations are used in this manuscript:

WRMSD	Work-Related Musculoskeletal Disorder
GUI	Graphical User Interface
MSD	Musculoskeletal Disorder
AI	Artificial Intelligence
DL	Deep Learning
ML	Machine Learning
IMU	Inertial Measurement Unit
OWAS	Ovako Working Posture Analysing
LUBA	Postural Loading Upper Body Assessment
RULA	Rapid Upper Limb Assessment
REBA	Rapid Entire Body Assessment
EAWS	Ergonomic Assessment Worksheet
MoCap	Motion Capture
CNN	Convolutional Neural Network
LSTM	Long Short-Term Memory
AWBA	Agricultural Whole-Body Assessment
AULA	Agricultural Upper-Limb Assessment
ALLA	Agricultural Lower-Limb Assessment
EMG	Electromyography
PUEU	Perceived Usefulness and Ease of Use
INUIT	Interface Usability Instrument
SUMI	Software Usability Measurement Inventory
